# Mutant Transcriptome Sequencing Provides Insights into Pod Development in Peanut (*Arachis hypogaea* L.)

**DOI:** 10.3389/fpls.2017.01900

**Published:** 2017-11-09

**Authors:** Liyun Wan, Bei Li, Yong Lei, Liying Yan, Xiaoping Ren, Yuning Chen, Xiaofeng Dai, Huifang Jiang, Juncheng Zhang, Wei Guo, Ao Chen, Boshou Liao

**Affiliations:** ^1^Key Laboratory of Biology and Genetic Improvement of Oil Crops, Ministry of Agriculture, Oil Crops Research Institute of Chinese Academy of Agricultural Sciences, Wuhan, China; ^2^Institute of Food Science and Technology of Chinese Academy of Agricultural Sciences, Beijing, China; ^3^Zhanjiang Academy of Agricultural Sciences, Zhanjiang, China

**Keywords:** peanut (*Arachis hypogaea* L.), pod width, lignin, RNA-seq, auxin

## Abstract

Pod size is the major yield component and a key target trait that is selected for in peanut breeding. However, although numerous quantitative trait loci (QTLs) for peanut pod size have been described, the molecular mechanisms underlying the development of this characteristic remain elusive. A peanut mutant with a narrower pod was developed in this study using ethyl methanesulfonate (EMS) mutagenesis and designated as the “pod width” mutant line (*pw*). The fresh pod weight of *pw* was only about 40% of that seen in the wild-type (WT) Zhonghua16, while the hull and seed filling of the mutant both also developed at earlier stages. Pods from both *pw* and WT lines were sampled 20, 40, and 60 days after flowering (DAF) and used for RNA-Seq analysis; the results revealed highly differentially expressed lignin metabolic pathway genes at all three stages, but especially at DAF 20 and DAF 40. At the same time, expression of genes related to auxin signal transduction was found to be significantly repressed during the *pw* early pod developmental stage. A genome-wide comparative analysis of expression profiles revealed 260 differentially expressed genes (DEGs) across all three stages, and two candidate genes, *c26901_g1* (*CAD*) and *c37339_g1* (*ACS*), responsible for pod width were identified by integrating expression patterns and function annotation of the common DEGs within the three stages. Taken together, the information provided in this study illuminates the processes underlying peanut pod development, and will facilitate further identification of causal genes and the development of improved peanut varieties with higher yields.

## Introduction

Peanut (*Arachis hypogaea* L.) is one of the most important oil crops in the world. Thus, as the demand for oil is ever-increasing, there is an urgent need to breed new peanut varieties with high yields, a characteristic that is dependent on pod size. Previous research has shown that pod size is mainly determined by quantitative trait loci (QTL), and several of these have been identified (Fonceka et al., 2012; Chen et al., 2016, 2017; Wang et al., 2016; Luo et al., 2017). Earlier work has shown that peanut genotypes from different backgrounds harbor distinct QTLs; thus, the primary characteristics of peanut which distinguish this plant from others include aerial flowering, a gynophore (peg) that elongates gravitropically, and subterranean fruiting. At the same time, swelling of the hull (also known as the shell) can influence potential yields. As a result of the rapid development of next generation sequencing technology, as well as the peanut genome project, significant progress in the study of this plant has been made in recent years (Bertioli et al., 2016; Chen et al., 2016b). Nevertheless, research on pod development lags far behind that of other cereal crops (Chen et al., 2013, 2016a). A typical peanut pod is comprised of three parts, a hull, a seed coat (or testa), and an embryo; of these, the hull forms a protective layer surrounding the seed, which itself functions to protect the endosperm and shield the embryo from external stresses. The hull of a peanut is composed of 46.8% holocellulose, 43.4% Klason lignin, 5.8% ash, and 4.0% organic solvent extracts (OSE) (Wang et al., 2016).

Lignin is a highly complex and heterogeneous polymer (Mellerowicz et al., 2001), a major component of the secondary wall of fibers and xylem cells. Lignification confers mechanical support, enables the transmission of water and solutes, and functions to protect plants against environmental stresses (Boerjan et al., 2003). Lignin is formed via the phenylpropanoid pathway through the oxidative polymerization of monolignols—predominantly coniferyl, *p*-coumaryl, and sinapyl alcohols. Thus, lignin polymer precursors include guaiacyl (G), *p*-hydroxyphenyl (H), and syringyl (S) subunits (Anterola and Lewis, 2002). In angiosperms, G and S subunits are the main components of lignin polymers, whereas H components are present in minor amounts (Vanholme et al., 2012). In addition to the predominant monolignols, hydroxycinnamic acids and hydroxycinnamaldehydes are also incorporated into lignin in smaller amounts (Baucher et al., 1996; Sibout et al., 2005; Ralph et al., 2008), along with other naturally occurring monomers such as caffeyl alcohol, dihydrohydroxycinnamyl alcohols, hydroxybenzaldehydes, and the flavonoid tricin; in addition, various acylated (i.e., by acetate, *p*-coumarate, ferulate, and *p*-hydroxybenzoate) monolignols can be incorporated into lignin polymers to varying extents in specific tissues and certain species (Boerjan et al., 2003; del Rio et al., 2012; Wilkerson et al., 2014). Lignin polymers function alongside wall polysaccharides to determine the structural integrity of the cell wall and also make certain cell types water-impermeable (Boerjan et al., 2003).

Most of the enzymes that function during lignin biosynthesis have been identified and are well-characterized (Fraser and Chapple, 2011). Reduced expression of cinnamyl alcohol dehydrogenase (CAD), for example, leads to the generation of unusual lignin polymers that are partially derived from hydroxycinnamaldehyde subunits (Kim et al., 2003, 2004), while similarly decreased expression of the 4-coumarate 3-hydroxylase gene (*C3H*) results in elevated levels of H lignin in plants (Hoffmann et al., 2004; Besseau et al., 2007; Bonawitz et al., 2014). Similarly, down-regulation of the ferulate 5-hydroxylase gene (*F5H*) eliminates, or reduces, the number of lignin S units content (Chapple et al., 1992; Reddy et al., 2005), while over-expression of *F5H* leads to an obvious increase in the number of these components (Meyer et al., 1998; Franke et al., 2000; Huntley et al., 2003; Stewart et al., 2009). Reduced caffeic acid O-methyltransferase (COMT) activity also stimulates the incorporation of 5-hydroxyconiferyl alcohol into lignin polymers (Ralph et al., 2001; Vanholme et al., 2010; Weng et al., 2010).

The crucial regulatory roles of plant hormones in controlling the process of lignification have been revealed in recent years. For example, the auxin-signaling double mutants *tir1* and *afb1* enable precocious endothecium lignification and stomium opening, while auxin controls the timing of dehiscence by down-regulating endothecium lignification via MYB26, and stomium opening via the modulation of jasmonic acid (JA) biosynthesis (Cecchetti et al., 2013). Over-expression of *YUC8* and *YUC9* leads to a marked lignification of aerial tissues in plants, while strong secondary growth is clearly related to elevated production of ethylene (Hentrich et al., 2013). Thus, ethylene and ERF (ethylene response factor) factors have been shown to play important roles in plant lignification (Prasad and Cline, 1987; Huang et al., 2013; Guo et al., 2016); ethylene negatively regulates the growth and elongation of primary roots via increased lignification of the cell wall and shorter root length (Huang et al., 2013). The cotton ethylene response-related factor (GbERF1) functions as an activator of *PAL, C4H, C3H, HCT, CCoAOMT, CCR*, and *F5H* genes, which in turn enhances lignin synthesis and substantially contributes to *Verticillium dahliae* resistance in plants (Guo et al., 2016).

A limited number of studies are available that provide information on the developmental process and regulation of peanut pods. Given the fact that these plants possess the unique characteristics of “aerial flowers and subterranean fruit,” the genes responsible for pod development are likely distinct from those in model plants such as *Arabidopsis* and rice. In this study, we identified an ethyl methanesulfonate (EMS) mutant with a narrower pod (seed width) (*pw*) than the Zhonghua16 wild-type (WT), and performed transcriptome profiling of both lines at three key pod developmental stages. The RNA-Seq data presented in this paper enhances our understanding of the transcriptome dynamics of peanut pod development and reveals the genes that are responsible for determining pod size.

## Materials and methods

### Plant materials and RNA isolation

The *pw* mutant used in this study was isolated from an EMS-induced population that originates from the Chinese elite cultivar Zhonghua 16, a WT that possesses both high yield and oil content characteristics. Both WT and *pw* (M6–M7 generations) lines were planted in the same areas (Wuhan, Yangluo, and Zhanjiang, China) between 2014 and 2016, and pod samples for further experiments were collected 20, 40, and 60 days after flowering (DAF), in 2015 from 12 individual plants. We then selected 12 representative pods from both the WT and *pw* mutant for each biological library construction (three biological replicates for each time point); total pod samples from both lines were sliced, immediately frozen in liquid nitrogen, and held at −80°C for subsequent RNA extraction with a Tiangen extraction kit (DP432). Three biological replicates were performed in all cases.

### Trait measurements

Pod and seed traits were investigated by applying previously published standard procedures (Jiang et al., 2006). The measurements taken include pod length and width, as well as seed length and width, and pod and seed weight at different developmental stages. Data from the Wuhan (2015 and 2016) and Yangluo (2015) planting sites was used for phenotypic characterization of the *pw* mutant (three biological replicates); a total of 20 representative pods from 10 individual plants were selected for each biological experiment from both lines.

### Sample preparation and microscopic observations

Peanut pods were collected at DAF 20, DAF 30, DAF 40, DAF 50, DAF 60, and DAF 70, and immediately fixed for 24 h at 4°C in a solution containing 5% formaldehyde, 5% acetic acid, and 50% ethanol. Pods were then dehydrated at intervals of 60 min through a 20% step-graded series of ethanol-water mixtures that terminated in 100% ethanol. Samples were then processed at 60-min intervals with a 30% step-graded series of ethanol-tert-butyl alcohol (TBA) that ended with 100% TBA, before being infiltrated with saturated paraffin-TBA mixtures for 24 h and embedded in paraffin for 48 h. Blocks were then polymerized completely at 4°C, and semi-thin sections between 5 and 8 μm in thickness were cut with a microtome blade KD-P (Zhejiang Jinhua Kedi Instrumental Equipment Co., Ltd, China), stained with safranin O/fast green, and visualized with a Nikon ECLIPSE TI-SR microscope (Nikon Instruments, Japan). Image J software was explored to measure cell size of the parenchymal cell.

### Quantification of lignin content

Cell walls were isolated from the hulls from six different *pw* and WT developmental stages via sequential extraction with three different solvents, comprising sodium phosphate (0.1 M, pH 7.2) at 37°C, 70% ethanol at 80°C (repeated more than three times), and acetone at room temperature. Lignin content was then measured by the acetyl bromide method; in short, between 2 and 5 mg of cell wall extract was dissolved in a 2.5 mL acetyl bromide:acetic acid (1:3, v/v) solution at room temperature for 24 h. This mixture was then transferred to a 10 mL volumetric flask, and 0.35 mL of 0.5 M hydroxylamine hydrochloride was added and the flask was filled with acetic acid to volume. Absorbance was then measured at 280 nm (Fukushima and Kerley, 2011).

### RNA-Seq, data processing, and gene annotation

As discussed, pod samples from *pw* and WT lines were harvested in 2016 at DAF 20, DAF 40, and DAF 60 and were subjected to RNA sequencing using an Illumina HiSeqTM2500 platform (Novogene, Beijing, China). A 3-μg sample of total RNA from each pod sample was then used to enrich messenger RNA and to construct complementary DNA libraries. High-quality reads (i.e., clean reads) were acquired by cutting adaptor sequences and removing low-quality reads with ambiguous nucleotides; all sequence data was uploaded into the BioProject database hosted by the National Center for Biotechnology Information (NCBI) under the BioSample accessions SAMN07437075 and SAMN07437076 (BioProject ID: PRJNA396814). Transcripts were then assembled using Trinity software (Grabherr et al., 2011), and gene expression was calculated by applying the fragments per kilobase per million reads (FPKM) method in the software RSEM (Li and Dewey, 2011). Gene function was then annotated via comparisons with multiple databases including non-redundant protein sequences (NCBI Nr), non-redundant nucleotide sequences (NCBI Nt), clusters of orthologous groups of proteins (KOG/COG), protein family (Pfam), the KEGG ortholog database (KO), the manually annotated and reviewed protein sequence database Swiss-Prot, and gene ontology (GO). Subsequent GO enrichment analysis of DEGs was implemented in R using the Goseq package and based on a Wallenius non-central hyper-geometric distribution (Young et al., 2010), adjusting for gene length bias in DEGs. The software KOBAS (Mao et al., 2005) was used to enrich KEGG pathways of DEGs, while the software packages Samtools (v0.1.18) and Picard-tools (v1.41) were used to sort, remove duplicate reads, and merge the bam alignment results of each sample.

### Quantitative reverse-transcription PCR (qRT-PCR) analysis

Reverse transcriptions were performed using an Invitrogen SuperScript Reagent Kit. We performed qRT-PCR using a Bio-Rad IQ5 qRT-PCR detection system (Bio-Rad, Hercules, CA, USA) and the SYBR® Premix ExTaq® (TAKARA). Gene expression of both lines (*pw* and WT) was detected for pod samples at DAF 20, DAF 40, and DAF 60, and three repeats of each reaction for individual genes were performed. The relative expression of each gene among different samples was calculated by using the 2^−ΔΔCt^ method and normalized by using the internal reference actin gene. Thermal cycle parameters were 95°C for 30 s, followed by 40 cycles of 95°C for 10 s, and between 50 and 56°C for 25 s in a 20 μl volume.

### Statistical analysis

All statistical analyses reported in this paper comprised Student's *t-*tests, with the number of biological replicates (n) in each experiment denoted in corresponding figure legends. Values were considered statistically significant at the *P* < 0.05 level and very significant at the *P* < 0.01 level; gene expression was scaled using the FPKM Z-score based on a mean value of three biological replicates in a heatmap.

## Results

### Phenotypic variation in pod width between *pw* and WT lines

**Figure 1:**
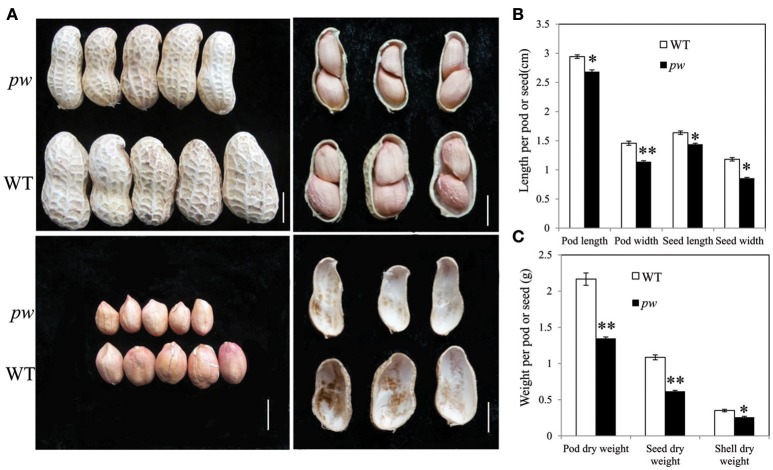
Phenotypic characterization of pod development in the *pw* and WT lines. **(A)** Peanut pod, seed, and hull phenotypes in *pw* and WT. **(B)** Pod and seed length and width of matured pod or seed of *pw* and WT. **(C)** Pod, seed, and hull dry weight of *pw* and WT. Statistically significant differences were analyzed according to three biological replicates (*t-*test; ^*^P < 0.05, ^**^P < 0.01). Values in **(B,C)** represent means ± SE (*n* = 3). Scale bars are 1 cm in **(A)**.

In order to investigate differences between *pw* and WT, we compared dry weight, length, pod and seed widths, and hull weight. Results show that both dry weight and width of both pods and seeds were significantly lower (*P* < 0.05) than those of WT after harvest (Figure 1A), while the pod and seed widths decreased from 1.46 to 1.08 cm in WT to 1.14 and 0.86 cm in *pw*, respectively (Figure 1B). At the same time, the dry weight of the pod, seed, and hull decreased from 2.17, 1.09, and 0.35 g in WT to 1.35, 0.62, and 0.26 g in *pw*, respectively (Figure 1C), but no significant differences were detected in other agronomic traits (Figure S1).

**Figure 2:**
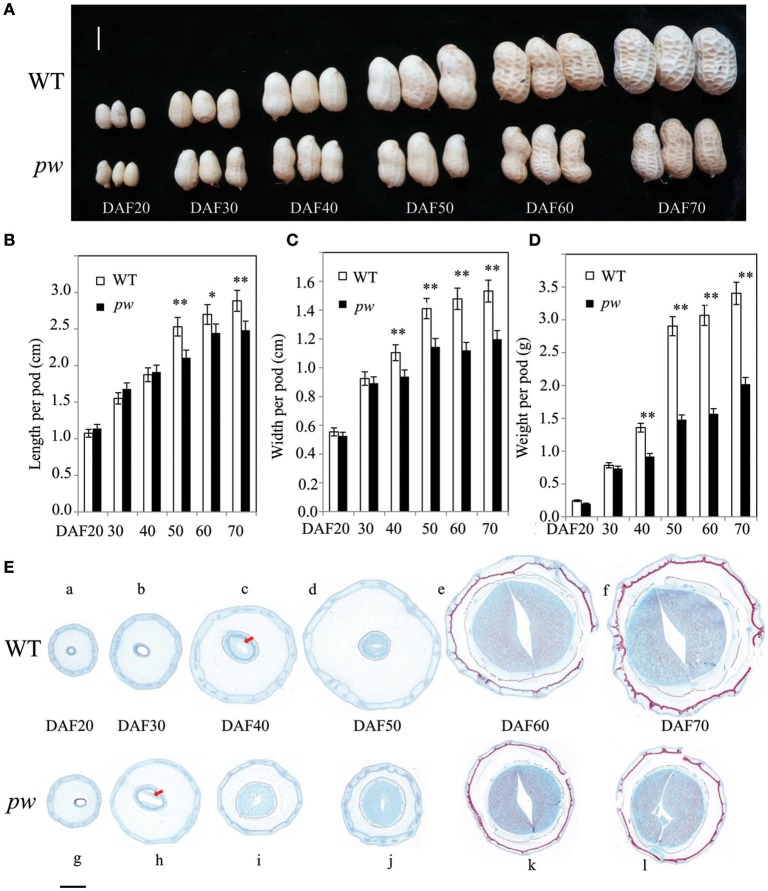
Development processes differ between the *pw* and WT lines. **(A)** Phenotypic characterization of six different developmental stages in *pw* and WT pods. **(B)** Pod length of *pw* and WT lines at six different developmental stages. **(C)** Pod width of *pw* and WT at six different developmental stages. **(D)** Pod weight of *pw* and WT at six different developmental stages. **(E)** Pod histochemical analysis of *pw* and WT at six different developmental stages. Significant differences were analyzed on the basis of three biological replicates (*t-*test: ^*^P < 0.05; ^**^P < 0.01). Values in **(B–D)** represent means ± SE (*n* = 3). Scale bars are 1 cm in **(A)** and 2,000 μm in **(E)**. The arrows indicate embryos.

In order to determine the precise pod developmental stage affected by mutation, we compared pod size, length, width, and fresh weight at six stages. Although, results show no difference in pod size between *pw* and WT at DAF 20 and DAF 40, the pod length of the *pw* was 2.1 cm, 15% shorter than the WT (2.5 cm) at DAF 50, an increase that continued until DAF 60 and DAF 70 (Figures 2A,B). Similarly, pod width was not significantly different between the *pw* and WT at DAF 20 and DAF30, but differences were seen at DAF 40 which were maintained to DAF 70 (Figure 2C). The *pw* pod fresh weight was also less than that of the WT at DAF 40, 0.8 g compared to 1.36 g, and this difference increased at DAF 50, DAF 60, and DAF 70 (Figure 2D). This phenotypic variation between the *pw* and WT lines suggests that the decrease in mutant pod weight was mainly the result of reductions in pod and seed size.

Significant differences in pod phenotype between *pw* and WT were found in this study. We therefore speculate that significant differences in pod development might also be seen; thus, to test this hypothesis, a series of histological stain experiments were carried out in *pw* and WT lines at the same six developmental stages. Results show that no embryo was present in either line at DAF 20, was present only in *pw* at DAF 30 (Figure 2E), and was present in both at DAF 40. In the WT line, the pod kept expanding at very low speed while the seed continued developing fast from DAF 50 to DAF 70, while for *pw*, the pod and seed grew synchronously.

### Cell size and lignification accumulation can be used to differentiate *pw* and WT lines

**Figure 3:**
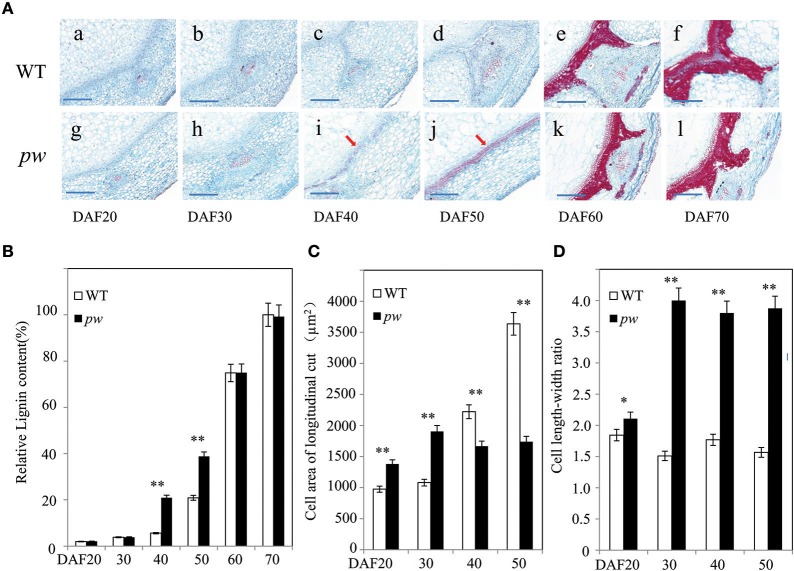
Differences in cell development and in the hull lignification process between the *pw* and WT lines. **(A)** Changes in lignin content and cell area during pod development in the *pw* and WT lines. **(a–f)** Detection and localization of lignin content at six different WT developmental stages. Scale bars are 200 μm. **(g–l)** Detection and localization of lignin content at six different *pw* developmental stages. Scale bars are 200 μm. **(B)** Relative lignin content of *pw* and WT lines at six different developmental stages. **(C)** Cell area of longitudinal cut in *pw* and WT lines at six different developmental stages. **(D)** Cell length–width ratio in *pw* and WT lines at six different developmental stages. Significant differences were analyzed on the basis of three biological replications (*t-*test: ^*^P < 0.05; ^**^P < 0.01). Values in **(B–D)** are means ± SE (*n* = 3). Scale bars are 200 μm in **(A)**. The arrows indicate lignified mesocarps.

As the results of this study reveal significant differences in the length, width, and weight of the pod and seed between the *pw* and WT lines, we further evaluated whether these differences are the result of cell size and wall components. Thus, to test this hypothesis, we measured cell area, length, and width, as well as the length–width ratio, and degree of lignification. The results of this investigation were consistent with expectations; transverse sections of developing pods revealed that at early seed developmental stages (i.e., DAF 20 and DAF 30), lignin staining was similar in the *pw* and WT lines (Figures 3Aabgh), while at DAF 40, the hull of *pw* had more lignin in middle-layer stereid bands than its counterpart (Figures 3Acdij). The distribution of lignin compounds was also similar again in the two late stages (Figure 3Aefkl), results that could be further confirmed by estimating Klason lignin contents using the method outlined by Fukushima and Kerley (2011). As in histochemical experiments, relative lignin contents were almost the same between the two lines in the two early stages (DAF 20 and DAF 30) before the *pw* lignin content increased to almost three times that of the WT. This marked difference was maintained until DAF 50 before it disappeared in the last two stages (Figure 3B); at DAF 20, the *pw* cell area in a longitudinal section was about 1,379 μm^2^, while it was just 976 μm^2^ in the WT. This difference between cell areas increased at DAF 30 and enlarged continuously in the WT until DAF 50, whereas in *pw* the increase in cell size ceased (Figure 3C). The cell length–width ratio was maintained at 1.7 in the WT and 3.8 in the *pw* line at DAF 30, DAF 40, and DAF 50 (Figure 3D).

### Transcriptome sequencing

**Figure 4:**
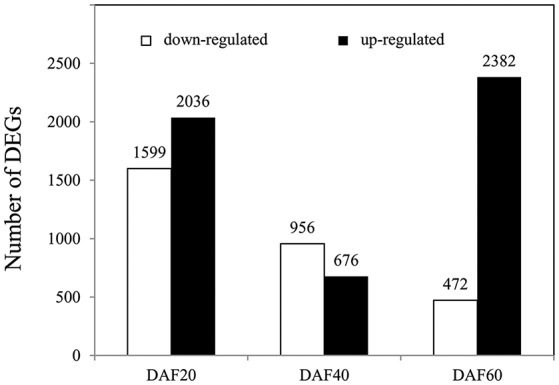
Number of DEGs in the *pw* and WT lines at DAF 20, DAF 40, and DAF 60. DEGs at DAF 20, DAF 40, and DAF 60 means *pw* expression relative to WT at each time point.

In order to determine the genes that underlie the regulation of lignin biosynthesis, metabolic pathways, and related hormone signaling, RNA-Seq was performed on whole pods at DAF 20, DAF 40, and DAF 60. To identify DEGs, a stringent value of FDR ≤ 0.001 and fold change ≥2 were used as thresholds; results show a total of 8,121 DEGs between the *pw* and WT lines (Table S1). A total of 3,635, 1,632, and 2,854 DEGs were identified at DAF 20, DAF 40, and DAF 60, respectively (Figure 4); at DAF 20, there were clearly more up-regulated genes (2,036) than down-regulated ones (1,599), while at DAF40, the number of up-regulated genes (676) was much less than down-regulated examples (956). Results revealed 472 up-regulated genes and 2,382 down-regulated ones at DAF 60 (Figure 4).

**Figure 5:**
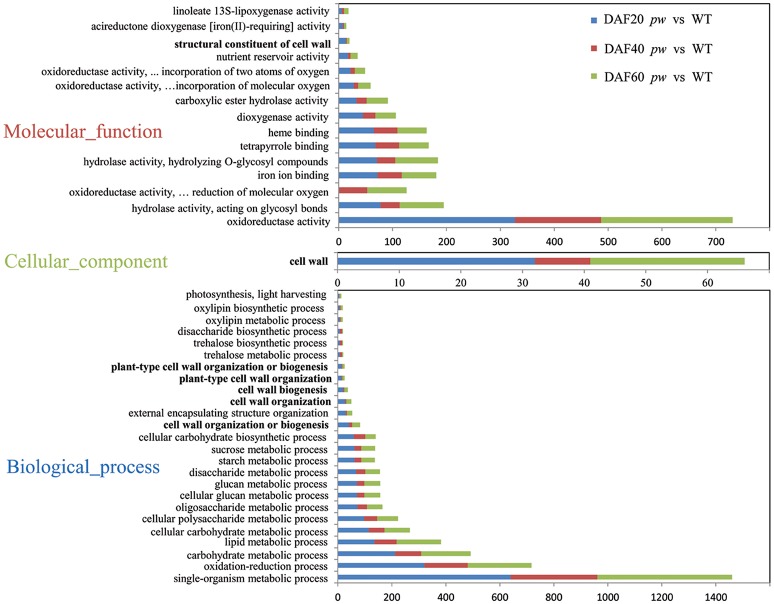
Functional categorization of DEGs between *pw* and WT lines. These genes were categorized using GO annotation.

In order to investigate the functional categories that were altered between *pw* and WT lines, GO categories were used to analyze DEGs. Although, the results of these comparisons (Figure 5) show that the GO-term distributions of DEGs correspond with biological processes, molecular function, and cellular components, the cell wall (i.e., 25 genes at DAF 20, nine genes at DAF 40, and 32 genes at DAF 60) was the only class annotated for the latter category (cellular components) (Figure 5). Data identified oxidoreductase (i.e., 327, 160, and 244), hydrolase acting on glycosyl bonds (i.e., 78, 35, and 82), oxidoreductase acting on paired donors with the incorporation, or reduction, of molecular oxygen (i.e., 1, 52, and 73), iron ion binding (i.e., 72, 45, and 64), and hydrolase hydrolyzing O-glycosyl compounds (i.e., 71, 34, and 79) as dominant molecular function categories (Figure 5). In terms of GO terms relating to biological processes, most DEGs were correlated with five major biological processes, including single-organism metabolism (i.e., 640, 321, and 499), oxidation-reduction (i.e., 321, 160, and 236), carbohydrate metabolism (i.e., 212, 97, and 183), lipid metabolism (i.e., 136, 82, and 164), and cellular carbohydrate metabolism (i.e., 115, 58, and 94) (Figure 5). An additional very considerable number of DEGs were correlated with cell wall organization or biogenesis (i.e., 41, 12, and 29; Figure 5).

Subsequent KEGG pathway analysis assigned the DEGs to 274, 249, and 269 metabolic pathways, respectively, associated with the three different developmental stages of the two lines. A total list of metabolic pathways is presented in Table S2; the top 30 metabolic and biological pathways identified in the *pw* line compared with the WT are listed in Table 1. These results show that phenylpropanoid biosynthesis was the most enriched pathway, followed by plant hormone signal transduction, starch, and sucrose metabolism, biosynthesis of amino acids, and carbon metabolism (Table 1, Table S2).

**Table 1:** The top 30 KEGG pathways in common between the *pw* and WT lines at DAF 20, DAF 40, and DAF 60.

**KEGG pathway**	**KEGG lD**	**Gene number**		
		**DAF20**	**DAF40**	**DAF60**
**Phenylpropanoid biosynthesis**	**ko00940**	**39**	**20**	**35**
**Plant hormone signal transduction**	**ko04075**	**45**	**19**	**30**
**Starch and sucrose metabolism**	**ko00500**	**35**	**18**	**35**
**Biosynthesis of amino acids**	**ko01230**	**39**	**14**	**19**
**Carbon metabolism**	**ko01200**	**36**	**15**	**17**
Phenylalanine metabolism	ko00360	34	15	17
Plant-pathogen interaction	ko04626	18	13	22
Amino sugar and nucleotide sugar metabolism	ko00520	21	10	21
Photosynthesis	ko00195	28	4	18
Protein processing in endoplasmic reticulum	ko04141	14	9	20
Endocytosis	ko04144	16	7	18
Glycolysis*I*Gluconeogenesis	ko00010	23	9	8
Alanine, aspartate, and glutamate metabolism	ko00250	15	7	14
Cysteine and methionine metabolism	ko00270	18	5	13
Oxidative phosphorylation	ko00190	17	14	5
Arginine and proline metabolism	ko00330	17	7	9
RNA transport	ko03013	1 8	4	11
Pentose and glucuronate interconversions	ko00040	17	3	12
Purine metabolism	ko00230	15	5	12
Peroxisome	ko04146	12	9	11
PI3K-Akt signaling path way	ko04151	14	8	10
Insulin signaling pathway	ko04910	13	8	11
Huntington's disease	ko05016	17	11	4
Carbon fixation in photosynthetic organisms	ko00710	16	9	6
Fatty acid metabolism	ko01212	12	3	16
Neurotrophin signaling pathway	ko04722	9	6	16
Non-alcoholic fatty liver disease (NAFLD)	ko04932	16	11	4
Influenza A	ko05164	10	7	14
Cyanoamino acid metabolism	ko00460	10	6	14

*Bold means top five KEGG related to phenotype*.

### Verifying DEGs during pod development

**Figure 6:**
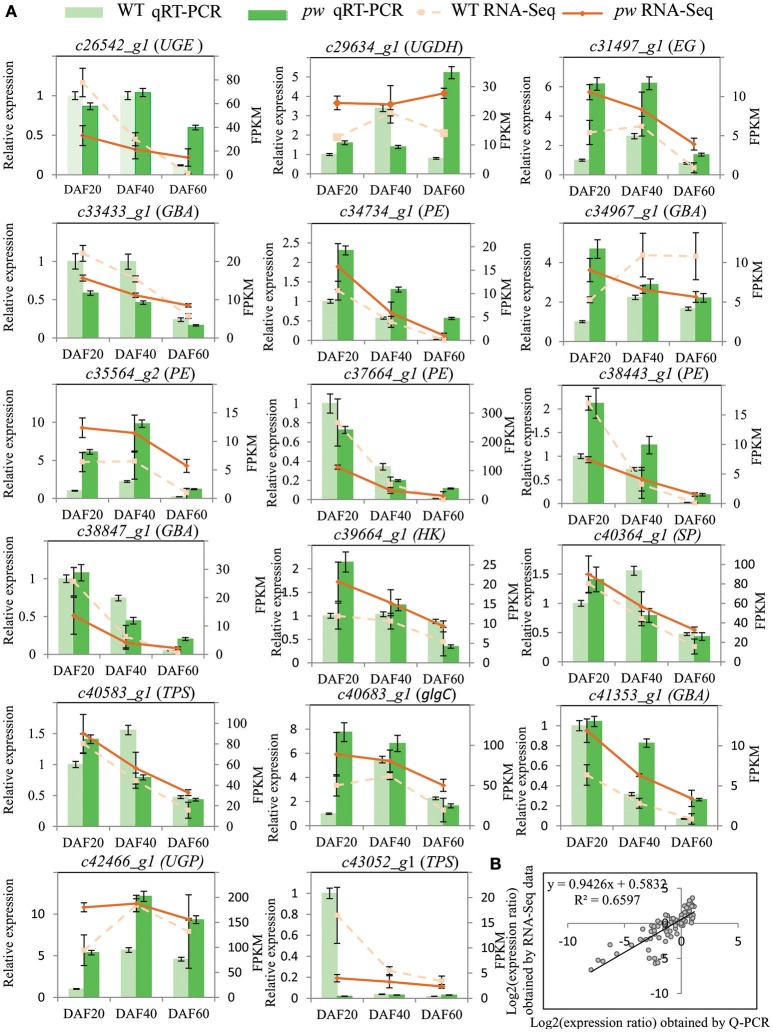
qRT-PCR verification of DEGs between *pw* and WT lines. **(A)** Transcript levels of 17 genes with average FPKM value ≥2. The y-axis shows the relative gene expression levels analyzed with qRT-PCR and RNA-Seq. Results of WT qRT-PCR (green columns) and *pw* qRT-PCR (dark green columns) correspond with qRT-PCR expression data, while WT RNA-Seq (broken lines) and *pw* RNA-Seq denote RNA-Seq data. Data are means of three repeats in all cases, and error bars represent SE (*n* = 3). **(B)** Comparison of gene expression ratios from qRT-PCR and RNA-Seq data. UGE, UDP-glucuronate 4-epimerase; UGDH, UDPglucose 6-dehydrogenase; EG, endoglucanase; GBA, beta-glucosidase; PE, pectinesterase; SP, starch phosphorylase; TPS, trehalose 6-phosphate synthase; glgC, glucose-1-phosphate adenylyltransferase; UGP, UTP–glucose-1-phosphate uridylyltransferase. The RNA-Seq log2 value of the expression ratio (y-axis) has been plotted against the three different developmental stages considered in this study (x-axis).

In order to evaluate the accuracy of the RNA-Seq data generated in this study, DEGs were verified via a biologically independent experiment that applied quantitative reverse-transcription PCR (qRT-PCR). Thus, 17 genes with FPKM ≥ 2 were selected for confirmation (the gene-specific primers used for this analysis are listed in Table S3) in this way (Figure 6A). Results of a linear regression analysis revealed an overall correlation coefficient of *R* = 0.81, indicative of a strong correlation between the transcription profiles revealed by RNA-Seq and abundances assayed using qRT-PCR (Figure 6B).

### Mutation of *pw* influences carbon (C) and nitrogen (N) metabolism

The supply of C and N to a growing seed will influence filling duration (Munier-Jolain et al., 2008). Therefore, as the KEGG enrichment analysis reported in this study revealed a large number of genes enriched in C and N metabolism (Table 1), we hypothesized that variation in their expression might be in response to differences in the pod-filling process between the two lines. In order to test this hypothesis, we investigated DEGs from the starch and sucrose metabolism pathway and identified 35, 18, and 35 genes at DAF 20, DAF 40, and DAF 60, respectively, that include hexokinase, alpha-1, 4-galacturonosyltransferase, and beta-glucosidase (Table S4). Data show that 20 (out of a total of 35), eight (out of a total of 18), and 32 (out of a total of 35) DEGs were up-regulated in the *pw* mutant during the three developmental stages (Table S4); thus, in terms of C metabolism, the expression levels of 25 (out of a total of 36), 7 (out of a total of 15), and 16 (out of a total of 17) genes were increased at each developmental stage, respectively, while for the biosynthesis of amino acids (which includes N metabolism), the expression levels of 30 (out of a total of 39), 7 (out of a total of 14), and 15 (out of a total of 19) DEGs were up-regulated in *pw* at DAF 20, DAF 40, and DAF 60, respectively. These data clearly show that both C and N metabolism were enhanced at DAF 20 and DAF 60; relevant gene expressions were confirmed using qRT-PCR (Figure S2).

### The transcriptional regulation of hormone signaling transduction pathway genes during peanut pod development

**Figure 7:**
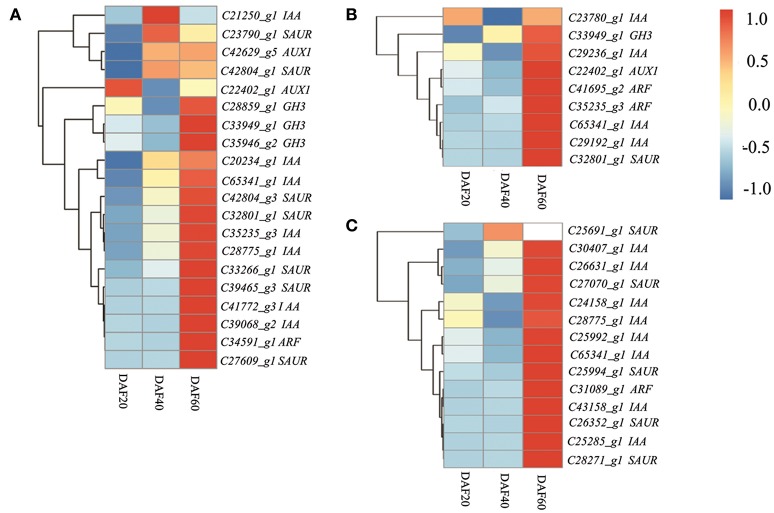
The auxin transduction pathway is depressed in the early developmental stages and activated at the late stage in the *pw* mutant. **(A)** Heatmaps represent the expression of the 20 DEGs in auxin pathway in the *pw* and WT lines at DAF 20. **(B)** Heatmaps represent the expression of the nine DEGs in auxin pathway in the *pw* and WT lines at DAF40. **(C)** Heatmaps represent the expression of the 14 DEGs in auxin pathway in the *pw* and WT lines at DAF 60. In the heatmap, gene expression was scaled using the Z-score of FPKM (mean value of three biological replicates). For each heatmap, the key is located at right side with FPKM values increasing from blue to red.

The KEGG analysis of RNA-Seq data presented in this paper provides evidence for a significant change in the expression of hormone signal transduction pathway genes in peanut pods, especially those involved in auxin signal transduction pathways. Previous studies have also shown that the signal transduction of hormones is important in the maturation process, and that the two mechanisms have a very close relationship (Hands et al., 2016; Nguyen et al., 2016). Thus, to gain deep insights into the transcriptional regulation of hormone signal transduction genes during peanut pod development, genes involved in this process were studied at the three different developing stages in the two lines. A total of 45 DEGs related to hormone signal transduction were identified at DAF 20, including 20 involved in auxin signal transduction, seven genes involved in brassinosteroid (BR) signal transduction, six involved in abscisic acid (ABA) signaling, six involved in gibberellin (GA) signal transduction, three involved in cytokinin (CTK) transduction, and one gene each involved in ethylene (ET), JA, and salicylic acid (SA) signal transduction. Results also revealed a lower number of DEGs involved in hormone transduction between the two lines at DAF 40, including nine involved in auxin signal transduction, three involved in JA signal transduction, two involved in ABA signal transduction, and one each involved in the BR, ET, SA, and CTK pathways. The dominant DEGs involved in hormone transduction remained those related to auxin signaling (14), almost half of the total (30) at DAF 60 (Table S5). Further analysis of auxin transduction-related genes revealed differential regulation between and the two strains, as the expression levels of those involved in this pathway were mostly down-regulated in the *pw* mutant at DAF 20 and DAF 40 compared with the WT line (Figure 7).

### Two candidate genes related to peanut pod size

In order to identify candidate genes that might be responsible for the *pw* pod phenotype, we further analyzed common DEGs from the three different developmental stages between the two lines. This analysis led to the identification of 260 DEGs between the two lines (Figure S3, Table S6); within the most common DEGs, two putative candidate genes, *c26901_g1* (*CAD*) and *c37339_g1* (*ACS*) were selected, both of which were significantly up-regulated in the *pw* mutant (Table S6). Results show that in the WT pod, the FPKM of the *c26901_g1* gene declined from 64.65 (DAF 20) to 36.88 (DAF 40) and fell further to 7.32 (DAF 60), while in the *pw*, the FPKM value this gene decreased from 104.13 (DAF 20) to 68.07 (DAF 40) and then fell further to 43.04 (DAF 60). At the same time, the expression level of the *c37339_g1* gene in the WT decreased from 2.84 (DAF 20) to zero (DAF 40 and DAF 60), while the expression level this gene in the *pw* line decreased from 7.86 (DAF 20) to 2.43 (DAF 40) and then fell further to 0.85 (DAF 60). Specifically, the gene *C26901_g1* encodes a CAD which is thought to be the key enzyme in lignin monomer synthesis; *CAD* genes in maize, *Medicago truncatula*, soybean, poplar, *Arabidopsis*, wheat, and pine have been shown to function in plant lignification and cell wall organization (Luderitz and Grisebach, 1981; Pillonel et al., 1991; Baucher et al., 1996; Lapierre et al., 2000; Sibout et al., 2005; Ma, 2010; Ma et al., 2011; Fornale et al., 2012; Zhao et al., 2013; Anderson et al., 2015). In contrast, the gene *C37339_g1* encodes for 1-aminocyclopropane-1-carboxylate synthase functions within the ethylene synthesis pathway to catalyze AdoMet conversion to 1-aminocyclopropane-1-carboxylic acid (ACC) and 5′-deoxy-5′-methylthioadenosine (MTA). This is important because ethylene is known to be involved in the lignification of plant secondary cell walls (Prasad and Cline, 1987; Zhong et al., 2002; Guo et al., 2016; Jin et al., 2017). However, whether, or not, the varied expression of these genes is responsible for the narrower pod width phenotype will need to be confirmed by further functional genomics studies.

## Discussion

Our current understanding of the role of the hull in peanuts is that this structure determines the size and shape of seed (Li et al., 2011; Huang et al., 2017). However, understanding of the molecular mechanisms that underlie pod size and shape remains limited; RNA-Seq was applied in this study to investigate transcriptome differences at three different developmental stages between *pw* and WT lines and thousands of DEGs were identified. In summary, lignification can be held responsible for differences in hull phenotypes between the *pw* and WT lines, while C and N metabolism were determined to be responsible for differences in seed filling. Both TFs and plant hormone signal transduction were shown to be responsible for the coordination of peanut pod development, and two genes (*c26901_g1*, encoding CAD, and *c37339_g1*, encoding ACS) were selected as candidates for controlling the *pw* mutation phenotype. The results of this study elucidated the peanut pod developmental process and will be useful in the future as a resource for the supply of genes and as a theoretical basis for developing improved peanut varieties with high yields.

### The *pw* mutation influences hull and seed developmental processes

Peanut hull size is one factor that influences seed size and shape. We therefore hypothesized that the narrower pod phenotype might result from the changes induced in the cell wall or to hormone signaling pathways. Initial observations of the *pw* line were carried out to test the composition of hulls and seeds from mature peanut pods, but the total lignin content of the hull, and the oil and protein content of the seed did not exhibit any variations. To investigate this further, we planted *pw* and WT lines at different locations and checked cell wall compositions and seed development patterns at the six developmental stages used throughout this research. Observations revealed that when the pod was opened, brittleness markedly differed between the two lines in the intermediate stages (i.e., DAF 40 and DAF 50). Subsequent quantification of cell wall components and histological staining experiments revealed that the lignin content varied at these two stages between the two lines; we also found that the seed enlarged along with the hull in the *pw* mutant, whereas the hull developed before the seed in the WT. These results clearly show that hull lignification and seed development conform to distinct patterns, phenomena that have also been observed in different peanut varieties with large and small pods (unpublished data). These hull lignification differences might be due to differences in lignin metabolic pathway expression; a faster seed developmental process in the *pw* line might be due, for example, to the accumulation of carbohydrates and proteins. We determined that the key modulator of both lignin and carbohydrate metabolism is related to higher regulatory networks, including TFs and hormone signaling pathways.

### Lignin metabolism is responsible for the narrow pod peanut phenotype

**Figure 8:**
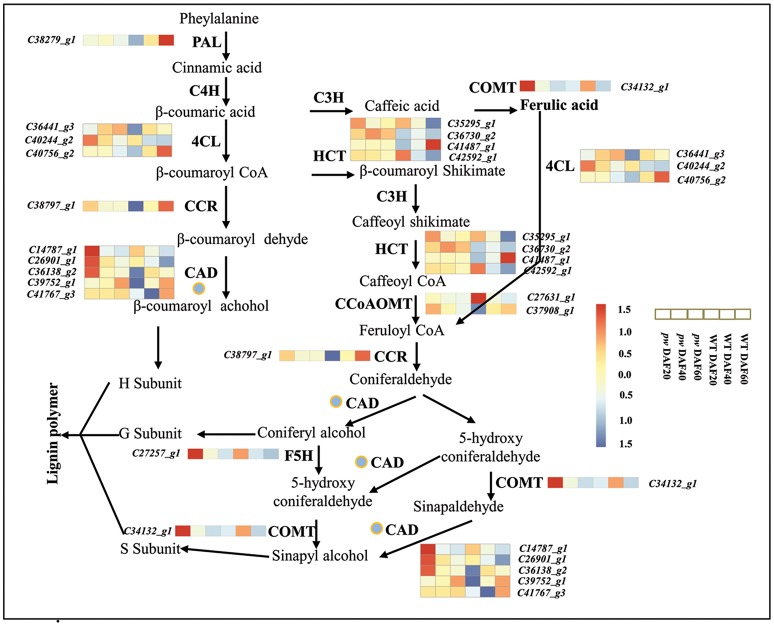
Lignin biosynthetic pathway. During early development, the hull of the *pw* mutant accumulates lignin earlier than that of the WT. PAL, phenylalanine ammonia lyase; C4H, cinnamate 4-hydroxylase; 4CL, 4-coumarate:CoA ligase; CCR, cinnamoyl-CoA reductase; CAD, cinnamyl alcohol dehydrogenase; C3H, 4-coumarate 3-hydroxylase; HCT, hydroxycinnamoyl-CoA:shikimate/quinate hydroxycinnamoyl transferase; CCoAOMT, caffeoyl-CoA 3-O-methyltransferase; COMT, caffeic acid O-methyltransferase; F5H, ferulate-5-hydroxylase. Gene expression was scaled using the Z-score of FPKM (mean value of three biological replicates) in the heatmap. For each heatmap, the key is located at right side with FPKM values increasing from blue to red.

Lignin is the second most abundant terrestrial biopolymer after cellulose and is formed by the oxidative polymerization of *p*-hydroxycinnamyl alcohol monomers on plant secondary cell walls (Boerjan et al., 2003). This material not only provides mechanical support, but also enables water and solutions to be transmitted from roots to leaves, and protects plants from environmental stimuli as an essential component of their growth and development (Bhuiyan et al., 2009; Zhao and Dixon, 2014; Barros et al., 2015). It is well known that monolignols are synthesized from phenylalanine through a series of enzymatic reactions catalyzed by phenylalanine ammonia lyase (PAL), cinnamic acid 4-hydroxylase (C4H), 4-coumarate: CoA ligase (4CL), cinnamoyl-CoA reductase (CCR), cinnamyl alcohol dehydrogenase (CAD), 4-coumarate 3-hydroxylase (C3H), hydroxycinnamoyl CoA: shikimate hydroxycinnamoyl transferase (HCT), caffeoyl-CoA O-methyltransferase (CCoAOMT), ferulic acid 5-hydroxylase (F5H), and caffeic acid O-methyltransferase (COMT) (Vanholme et al., 2008). The transcriptome data generated in this study demonstrate that the genes *PAL, C4H, 4CL, CCR, CAD, HCT, F5H, CCoAOMT*, and *COMT* are all activated in the early developmental stages (i.e., DAF 20 and DAF 40) in the *pw* mutant, and that *PAL, CCR*, and to some extent *4CL, CAD, HCT*, and *CCoAOMT*, exhibited higher levels of expression at DAF 60 in the WT as compared to the *pw* line (Figure 8, Table S7). These results suggest that the lignification process in the *pw* pod starts at an earlier stage than in the WT line. In addition, we show that *PAL, C4H, 4CL, CCR, CAD, HCT, F5H, CCoAOMT*, and *COMT* are all key genes underlying differences in peanut hull lignification processes between the two lines.

Previous work has shown that the suppression of PAL, C4H, 4CL, CCR, CAD, HCT, F5H, COMT, and CCoAOMT enzymes in higher plants affects their final lignin composition (Chapple et al., 1992; Meyer et al., 1998; Boerjan et al., 2003; Bhuiyan et al., 2009). We have also noted the fact that the lignin content of the *pw* line changed the lignification of the pod; this difference is related to the down-regulation of genes involved in the lignin synthesis pathway, including *CAD, F5H*, and *COMT*. These results imply that the mechanism of lignin synthesis in peanuts is similar to that seen in other plants.

The mechanisms of monolignol transport, lignin polymerization and deposition have all recently been discussed. Monolignols are thought to be moved by ABC transporters to the cell wall via the plasma membrane (Alejandro et al., 2012), and CASP-like proteins, NADPH oxidase, and peroxidase are all required for monolignol polymerization and deposition processes (Lee et al., 2013). Similarly, laccases (*LAC4, LAC11*, and *LAC17*) have also been shown to be important genetic regulators of lignification in *Arabidopsis* fiber cell walls (Berthet et al., 2011; Zhao et al., 2013), while the transcriptome analysis presented here shows that several structural genes involved in monolignol were up-regulated in the *pw* line in early developmental stages (Figure 8) and influenced lignification. At the same time, the expression levels of genes involved in both lignin polymerization and deposition were significantly different at DAF 40 between the two lines, including peroxidase- (especially the ones enriched during phenylpropanoid biosynthesis) and laccase-encoding genes (Tables S7, S8). These results demonstrate that variation in the process of lignification is not just the result of monolignol synthesis but is also due to the polymerization and deposition of this biomaterial.

### TFs and the signal transduction of plant hormones coordinate and regulate the development of the peanut pod

Over the last 10 years, MYB and NAC TFs have been shown to play essential roles in controlling secondary cell wall lignin deposition in *Arabidopsis* (Mitsuda et al., 2007; Zhong et al., 2007a,b, 2008; Mitsuda and Ohme-Takagi, 2008; Zhou et al., 2009; Agarwal et al., 2016). Thus, MYB TFs also play key roles in the regulation of compounds derived from phenylpropanoid synthesis in plants. For example, EgMYB1, MYB31/42, BpiMYB46, AtMYB61, and MdMYB93 have all been identified as regulators of lignification; these MYBs can bind directly to the promoter of most monolignol pathway genes and regulate their expression (Zhong et al., 2008; Agarwal et al., 2016; Legay et al., 2016; Scully et al., 2016; Guo et al., 2017; Soler et al., 2017). Previous work has also shown that the NAC TFs, AtVND6/7, NST1, NST2, and NST3 function in the upper stream of the transcriptional regulation cascade (regulating the expression of MYB factors) in secondary cell wall lignification and that over-expression of NSTs will result in elevated levels of lignification (Mitsuda et al., 2007; Mitsuda and Ohme-Takagi, 2008). Our analysis of common DEGs at the three developmental stages considered in this paper did not identify any MYB or NAC transcription factor-encoding genes, although a number involved in auxin and ethylene signaling pathways were identified; overall, this suggests that modulation of peanut pod lignification is distinct from that in known model plants.

Plant hormones and TFs play essential roles in pod and seed development. The RNA-Seq data presented in this study shows that hormone signaling pathway genes in the *pw* pod varied greatly during development when compared with the WT line, especially those involved in the auxin signal transduction pathway. This is interesting because the plant hormone auxin has been shown to play a role in lignin synthesis (Anterola and Lewis, 2002; Hentrich et al., 2013; Anderson et al., 2015); our transcriptome data showed that 18 (out of a total of 20) and eight (out of a total of nine) auxin signal transduction genes present in the KEGG pathway “plant hormone signal transduction,” were significantly down-regulated in the *pw* mutant at DAF 20 and DAF 40. This result implies that auxin signal transduction is strongly suppressed in the mutant, even though these genes were strongly up-regulated at DAF 60 (Figure 7). Expression differences in auxin transduction and lignin synthesis were the opposite of one another, consistent with previous studies (Brown et al., 2001; Besseau et al., 2007; Li et al., 2010; Steenackers et al., 2016), but the regulatory relationship between auxin and lignin will require further clarification in the future. Ethylene and ERF TFs have been reported to control the development of plant seeds and grains in various different species (Zhong et al., 2002; Hentrich et al., 2013; Huang et al., 2013; Guo et al., 2016). Based on the common DEGs seen at the three different stages, we identified four ethylene transcription factor-encoding genes (i.e., *c27008_g1, c25926_g1, c32892_g1*, and *c27924_g1*) that had extremely low levels of expression at DAF 20 and DAF 40 and higher levels of expression at a late stage (DAF 60) in the *pw* mutant. We identified one 1-aminocyclopropane-1-carboxylate synthase (*ACS*) gene involved in ethylene synthesis that had differences in expression during all three developmental stages.

**Figure 9:**
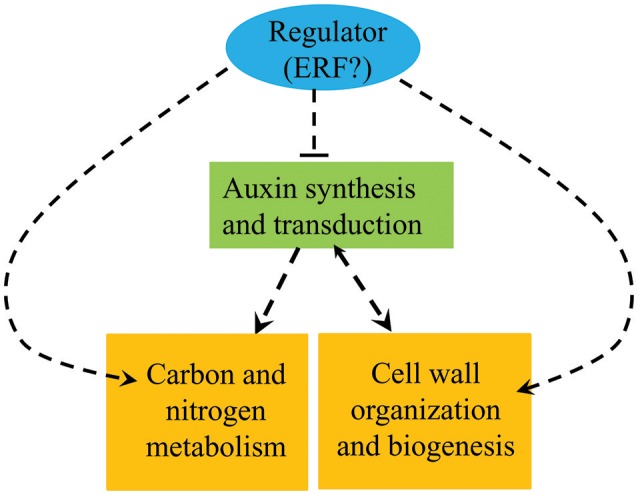
Summary of the biological pathways involved in peanut pod development. Orange boxes represent genes and proteins up-regulated in the *pw* mutant compared with the WT, while the green box represents genes and proteins down-regulated in the *pw* line compared with the WT.

The results presented here show that the auxin, ethylene, C/N metabolism, and lignin synthesis pathways all vary between *pw* and WT and can therefore be selected as important candidates controlling the development of peanut pods. We hypothesize that the ethylene and auxin signaling pathways interact cooperatively with one another, thereby modulating the expression of genes of lignin synthesis and those involved in C metabolism to influence the lignification process and the accumulation of carbohydrates and proteins during the early developmental stages. At the same time, enhanced C/N metabolism and lignification repress the auxin signaling pathway in the *pw* mutant (Figure 9). Further research is required, however, and in particular we have been unable to demonstrate the nature of the interactions between the four factors responsible for the narrowed pod and seed width trait in the *pw* mutant.

## Author contributions

Conceived and designed the experiments: LW, YL, LY, HJ, and BoL. Performed the experiments: LW and BeL. Analyzed the data: LW. Contributed reagents/materials/analysis tools: WG, XD, YL, LY, HJ, XR, YC, and AC. Wrote the paper: LW and BoL. All authors have read and approved the manuscript.

### Conflict of interest statement

The authors declare that the research was conducted in the absence of any commercial or financial relationships that could be construed as a potential conflict of interest.

## Supplementary material

Table S1 DEGs between the *pw* and WT lines at DAF 20, DAF 40, and DAF 60.

Table S2 KEGG pathway enrichment of DEGs between the *pw* and WT lines at DAF 20, DAF 40, and DAF 60.

Table S3 Gene-specific primers used in the qRT-PCR analysis.

Table S4 Expression and annotation of the DEGs involved in C and N metabolism. UGDH, UDPglucose 6-dehydrogenase; UGE, UDP-glucuronate 4-epimerase; GAUT, alpha-1,4-galacturonosyltransferase; EG, endoglucanase; GBA, beta-glucosidase; PE, pectinesterase; HK, hexokinase; FK, fructokinase.

Table S5 DEGs involved in hormone synthesis and signal transduction between the *pw* and WT lines.

Table S6 DEGs in common between the *pw* and WT lines at three different developmental stages.

Table S7 Expression and annotation of the DEGs involved in the lignin synthesis pathway.

Table S8 Expression of peroxidase and laccase encoding genes.

Figure S1 Developmental observations of *pw* and WT lines.

Figure S2 Results of qRT-PCR verification of changes in the expression of starch and sucrose metabolism pathway genes in *pw* compared with WT. The y-axis of this graph shows relative gene expression levels analyzed by qRT-PCR and RNA-Seq. The WT qRT-PCR (green columns) and *pw* qRT-PCR (dark green columns) correspond to qRT-PCR expression data, while WT RNA-Seq (broken lines) and *pw* RNA-Seq refers to RNA-Seq data. In all cases, the data presented are means of three repeats, and the error bars represent standard errors (*n* = 3).

Figure S3 Venn diagram showing the number of DEGs at the three different developmental stages.
