# Supplementary material for: Mutant Transcriptome Sequencing Provides Insights into Pod Development in Peanut (Arachis hypogaea L.)
Source: Front Plant Sci. 2017 Nov 9;8:1900. doi: 10.3389/fpls.2017.01900 (PMC5684126; doi:10.3389/fpls.2017.01900)
Supplement: Table S3 — Gene-specific primers used in the qRT-PCR analysis. [file Table3.DOC]

|  |  |
| --- | --- |
| Gene ID | Primer sequence |
| *c25713_g1*-qPCR-F | CTGATGCTTGAACCTGCCAATA |
| *c25713_g1*-qPCR-R | GTGTTTACTCTACAGCTGTGCA |
| *c26542_g1*-qPCR-F | GAAAGAAAGAGAGAATGCCGTCA |
| *c26542_g1*-qPCR-R | GATGAGGAACAAAGCCCAGAG |
| *c28608_g1*-qPCR-F | CAGCTGTGGAAGTTAGGAACAC |
| *c28608_g1*-qPCR-R | CAATGACTTCGGAACTTGGGAA |
| *c29634_g1*-qPCR-F | GGCAAATTCGAACCGTACCATA |
| *c29634_g1*-qPCR-R | AGAAATGAGATGGGGAGTGGTT |
| *c31497_g1*-qPCR-F | GGCCTGCTAGTATCGCTCATAA |
| *c31497_g1*-qPCR-R | GCTTGCTTGTGTTGGGTGTATA |
| *c32913_g1*-qPCR-F | CTGGTGGAACGGGTCTTATTTC |
| *c32913_g1*-qPCR-R | AGACACGCGTTACTGTAGAGAT |
| *c33433_g1*-qPCR-F | TGGGAGAAGTCTATTTCCGCTT |
| *c33433_g1*-qPCR-R | CACTATTCCTCCGCGAAGTTTT |
| *c34734_g1*-qPCR-F | GCCAAGTCAAATGAGTCCGTC |
| *c34734_g1*-qPCR-R | GCCTTCTATAATCCCTCCACCA |
| *c34734_g2*-qPCR-F | CCGTTCCTCAATCGAATTCCC |
| *c34734_g2*-qPCR-R | CACTGTGTTAGGCGACCAAC |
| *c34967_g1*-qPCR-F | TGGTTACGGGTCTTTGTTGTTT |
| *c34967_g1*-qPCR-R | CCAACACGTGCTAGTTCCTAAA |
| *c35297_g1*-qPCR-F | TAGGTTGTTGTTGTTGCTGAGG |
| *c35297_g1*-qPCR-R | GATTCTCCATTGACAAGCGTGA |
| *c35436_g1*-qPCR-F | GAACCGCACTAGATAGCATAGC |
| *c35436_g1*-qPCR-R | GCCGTTTCTCATCCTGTAAACA |
| *c35564_g2*-qPCR-F | CAAAACACTTGTGCAGGTAGTT |
| *c35564_g2*-qPCR-R | AGGGAAATCTTTGGTTACCTTCA |
| *c36605_g1*-qPCR-F | TGTTTAGCCTAAGAGTGAGGCA |
| *c36605_g1*-qPCR-R | GCTTGAGTTGCTTGTGAATTGT |
| *c37664_g1*-qPCR-F | GCAAGGCACAAGTTTATTCAGC |
| *c37664_g1*-qPCR-R | CTAATCCAAGGCGATGTTTGGT |
| *c38443_g1*-qPCR-F | AGCCCTTCCACTTGACTCTATT |
| *c38443_g1*-qPCR-R | TTTCAGGCTTTGGGAACTTGAG |
| *c38847_g1*-qPCR-F | AATTGATGTTTGTGTGCGTGTG |
| *c38847_g1*-qPCR-R | ACCTTCGAGTTCTGTTGAGAGT |
| *c39664_g1*-qPCR-F | TGGTAGGAGCATGTATCAGAAAA |
| *c39664_g1*-qPCR-R | CACTCTTCCTTCAATTTCTCGCT |
| *c40364_g1*-qPCR-F | GTGTGTACTGTGTTGGGTGTAG |
| *c40364_g1*-qPCR-R | CCTTTAGATAGGGCCTCTGGAG |
| *c40583_g1*-qPCR-F | TGTTCCTTTTCGTCCTCCCATA |
| *c40583_g1*-qPCR-R | GTACATGACCTCAGAACTTGGC |
| *c40683_g1*-qPCR-F | TAGCATCGGGATCAAGACAAGT |
| *c40683_g1*-qPCR-R | CCTCTCATTCTCCTCCTCACAA |
| *c41353_g1*-qPCR-F | ACAAGAAACACAGCAATTGGAGT |
| *c41353_g1*-qPCR-R | GCGCTAAGGACATGAACAATTC |
| *c42466_g1*-qPCR-F | CGTTGTTTATTTTGAGCCTGCC |
| *c42466_g1*-qPCR-R | GTGAGCAATTGACAAGGACTCA |
| *c43052_g1*-qPCR-F | GGAGTCCTGAACACTTGACTTG |
| *c43052_g1*-qPCR-R | TCACAGAACCATCACAACACAC |
