# Supplementary figures and images for: Mutant Transcriptome Sequencing Provides Insights into Pod Development in Peanut (Arachis hypogaea L.)
Source: Front Plant Sci. 2017 Nov 9;8:1900. doi: 10.3389/fpls.2017.01900 (PMC5684126; doi:10.3389/fpls.2017.01900)

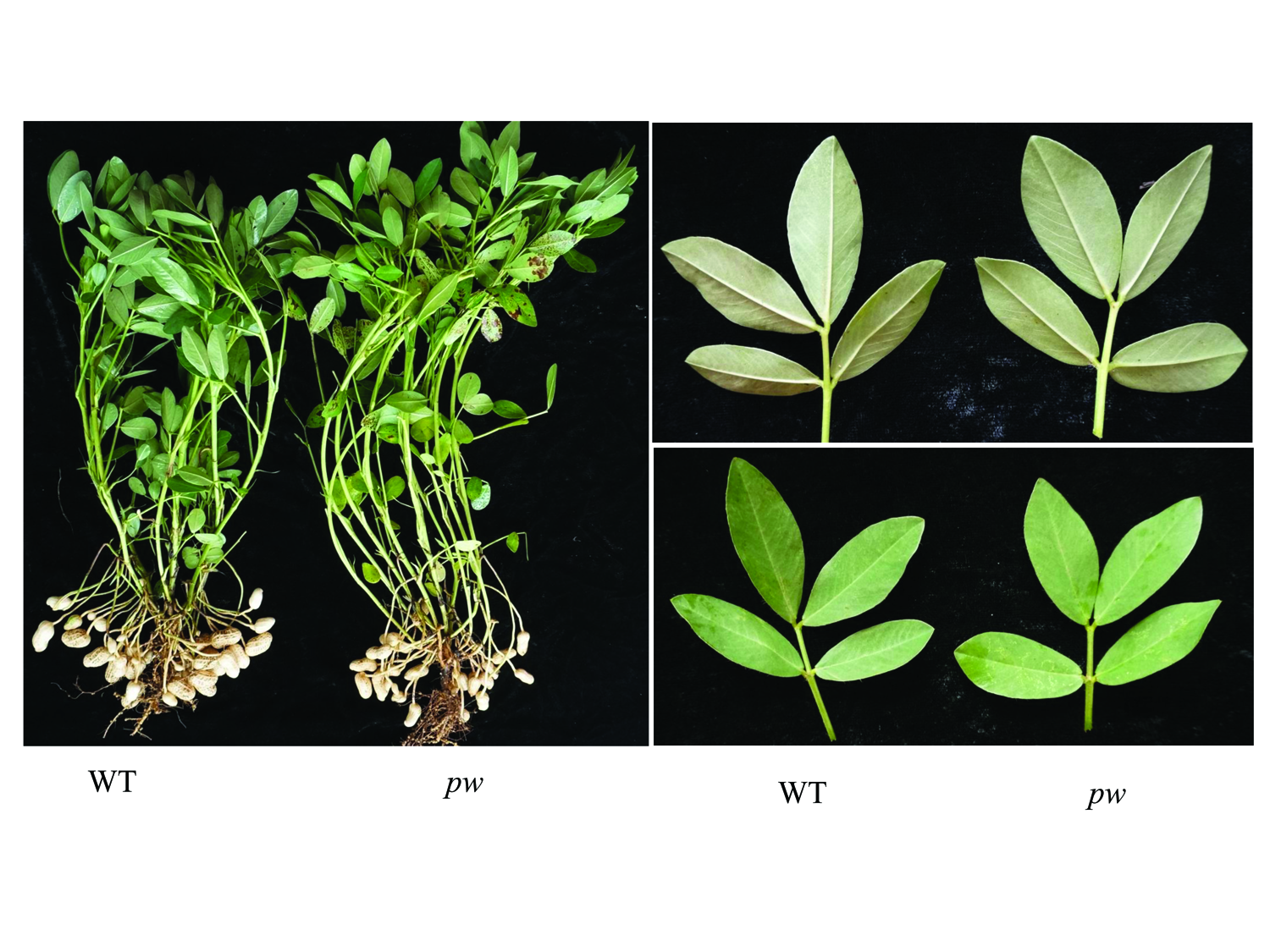

Supplement: Figure S1 — Developmental observations of pw and WT lines. [file Image1.TIF]

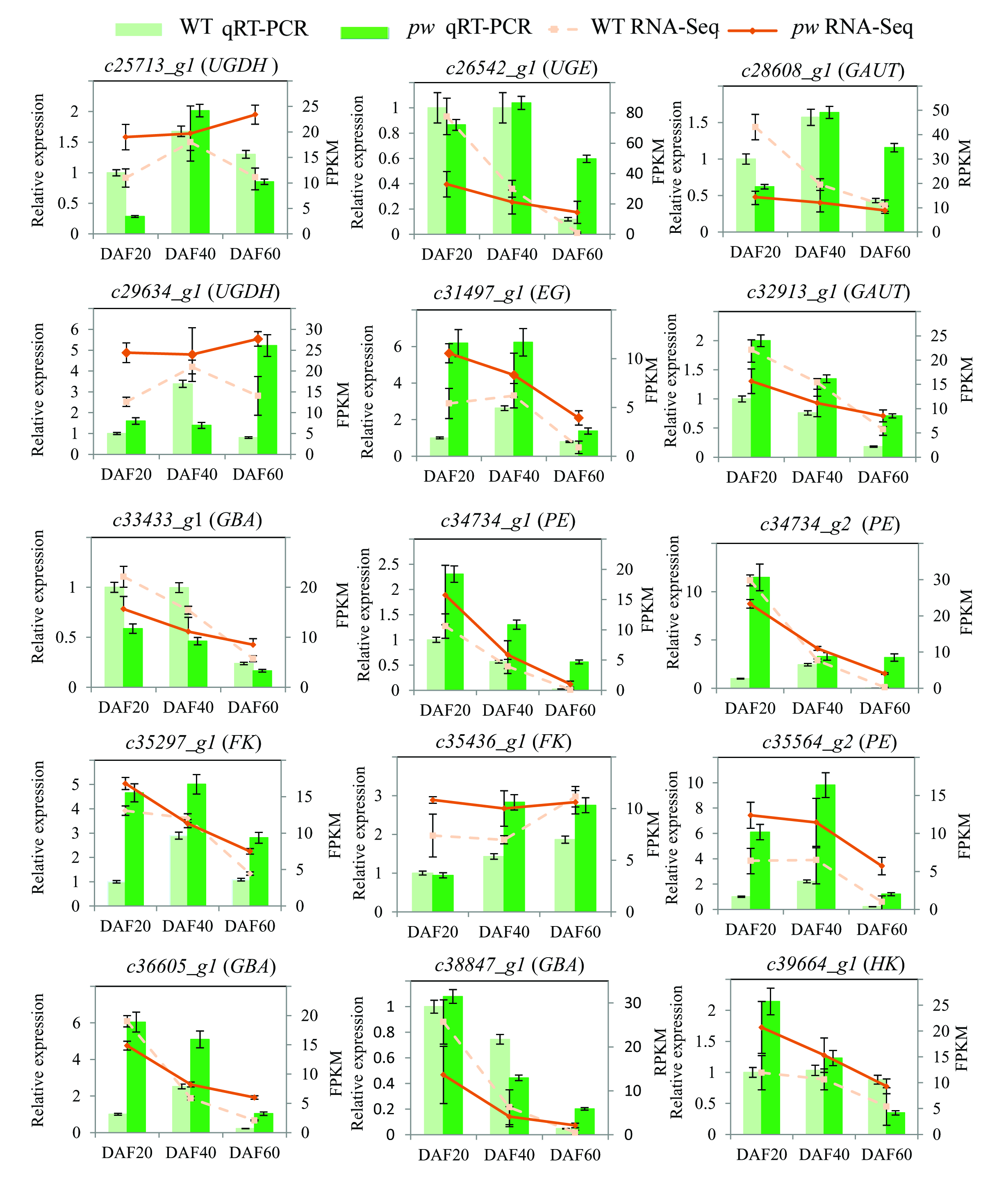

Supplement: Figure S2 — Results of qRT-PCR verification of changes in the expression of starch and sucrose metabolism pathway genes in pw compared with WT. The y-axis of this graph shows relative gene expression levels analyzed by qRT-PCR and RNA-Seq. The WT qRT-PCR (green columns) and pw qRT-PCR (dark green columns) correspond to qRT-PCR expression data, while WT RNA-Seq (broken lines) and pw RNA-Seq refers to RNA-Seq data. In all cases, the data presented are means of three repeats, and the error bars represent standard errors (n = 3). [file Image2.TIF]

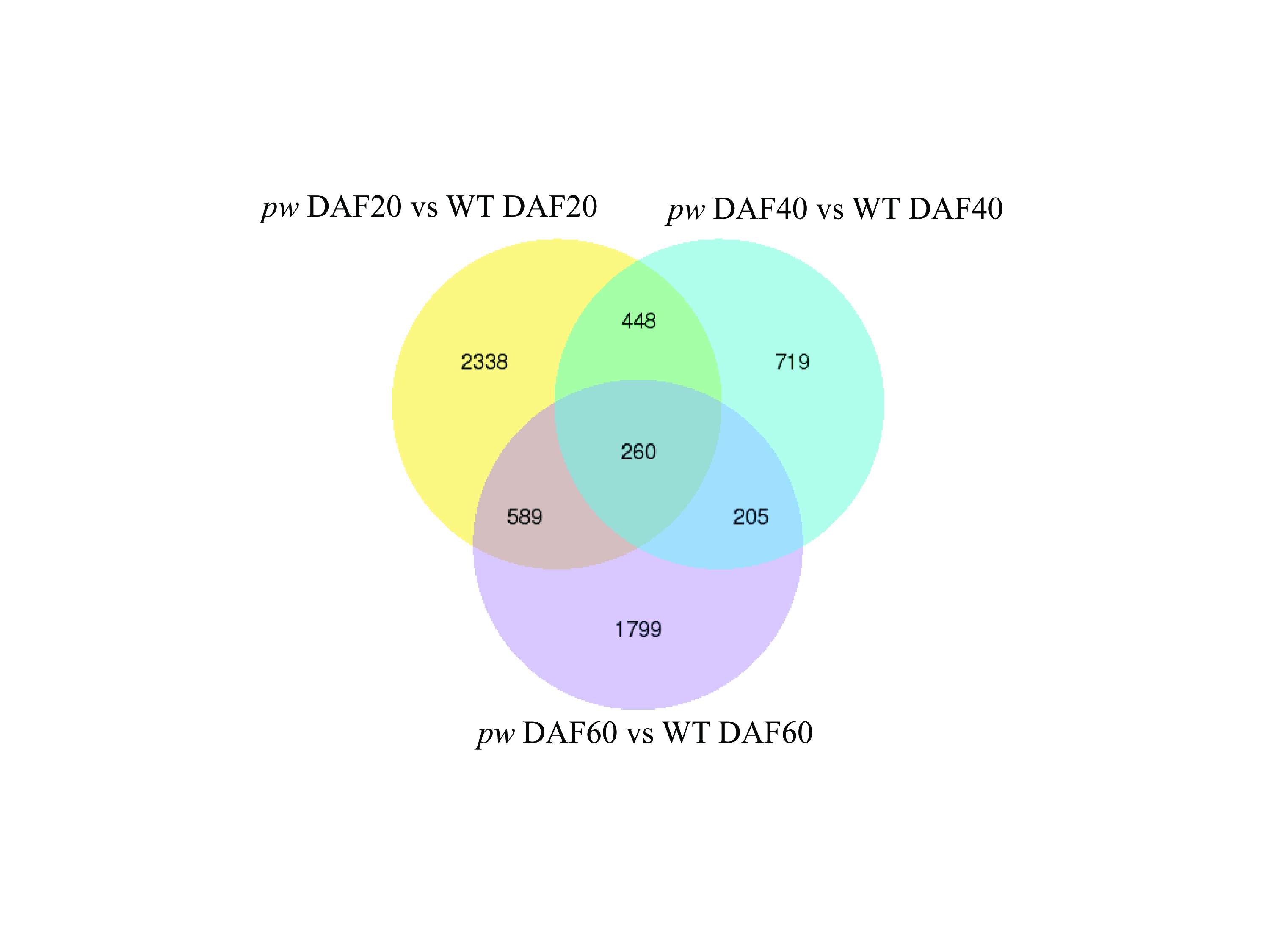

Supplement: Figure S3 — Venn diagram showing the number of DEGs at the three different developmental stages. [file Image3.TIF]
